# Neuroprotective and Antioxidant Role of Oxotremorine-M, a Non-selective Muscarinic Acetylcholine Receptors Agonist, in a Cellular Model of Alzheimer Disease

**DOI:** 10.1007/s10571-022-01274-9

**Published:** 2022-09-03

**Authors:** Domenico Nuzzo, Monica Frinchi, Costanza Giardina, Miriana Scordino, Mariachiara Zuccarini, Chiara De Simone, Marta Di Carlo, Natale Belluardo, Giuseppa Mudò, Valentina Di Liberto

**Affiliations:** 1 CNR, Istituto per la Ricerca e l’Innovazione Biomedica, Via Ugo La Malfa 153, 90146 Palermo, Italy; 2 Dipartimento di Biomedicina, Neuroscienze e Diagnostica Avanzata, Università di Palermo, corso Tukory 129, 90134 Palermo, Italy; 3 Dipartimento di Scienze Mediche, Orali e Biotecnologiche, Università di Chieti-Pescara, Via dei Vestini 29, 66100 Chieti, Italy; 4 Center for Advanced Studies and Technologies (CAST), Università di Chieti-Pescara, Via L. Polacchi, 66100 Chieti, Italy

**Keywords:** Oxidative stress, SH-SY5Y cells, Mitochondria, β-amyloid

## Abstract

Alzheimer disease (AD) is a multifactorial and age-dependent neurodegenerative disorder, whose pathogenesis, classically associated with the formation of senile plaques and neurofibrillary tangles, is also dependent on oxidative stress and neuroinflammation chronicization. Currently, the standard symptomatic therapy, based on acetylcholinesterase inhibitors, showed a limited therapeutic potential, whereas disease-modifying treatment strategies are still under extensive research. Previous studies have demonstrated that Oxotremorine-M (Oxo), a non-selective muscarinic acetylcholine receptors agonist, exerts neurotrophic functions in primary neurons, and modulates oxidative stress and neuroinflammation phenomena in rat brain. In the light of these findings, in this study, we aimed to investigate the neuroprotective effects of Oxo treatment in an in vitro model of AD, represented by differentiated SH-SY5Y neuroblastoma cells exposed to Aβ_1-42_ peptide. The results demonstrated that Oxo treatment enhances cell survival, increases neurite length, and counteracts DNA fragmentation induced by Aβ_1-42_ peptide. The same treatment was also able to block oxidative stress and mitochondria morphological/functional impairment associated with Aβ_1-42_ cell exposure. Overall, these results suggest that Oxo, by modulating cholinergic neurotransmission, survival, oxidative stress response, and mitochondria functionality, may represent a novel multi-target drug able to achieve a therapeutic synergy in AD.

**Graphical Abstract:**

Illustration of the main pathological hallmarks and mechanisms underlying AD pathogenesis, including neurodegeneration and oxidative stress, efficiently counteracted by treatment with Oxo, which may represent a promising therapeutic molecule. Created with BioRender.com under academic license.

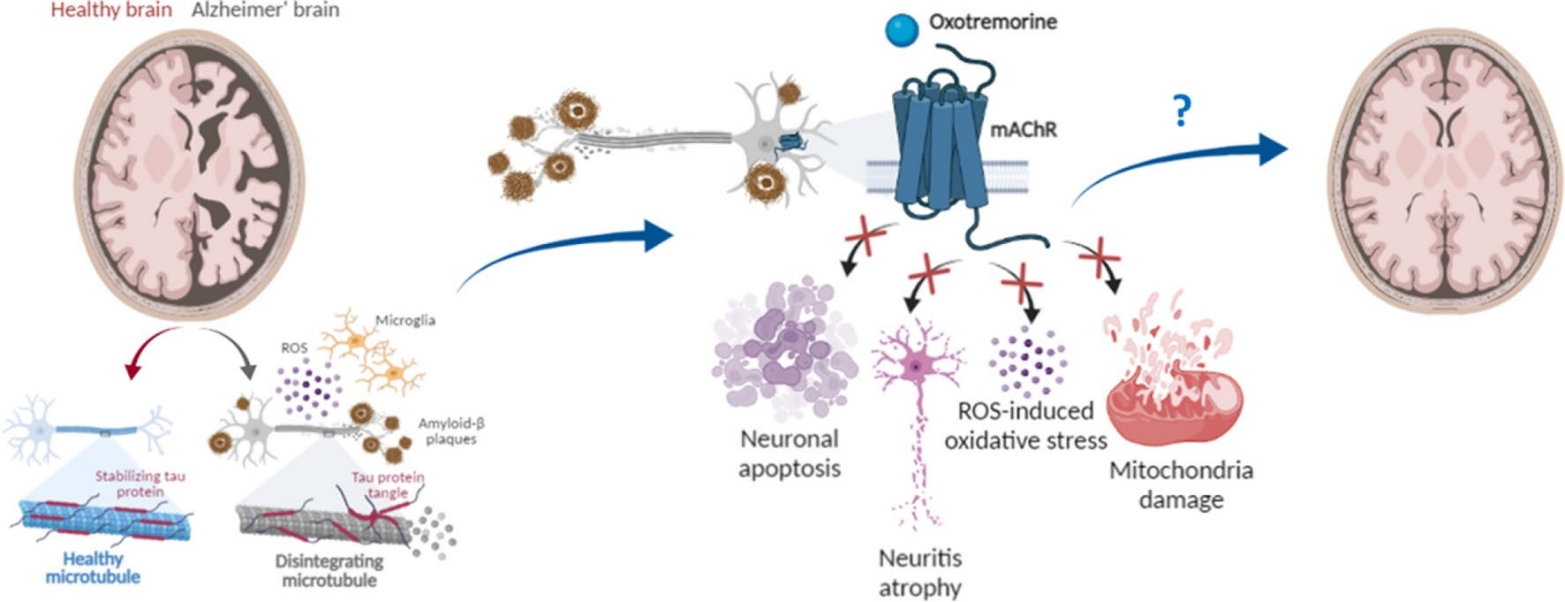

**Supplementary Information:**

The online version contains supplementary material available at 10.1007/s10571-022-01274-9.

## Introduction

Alzheimer disease (AD) is a progressive neurodegenerative disorder, and the most prevalent cause of dementia (AlzheimersDement 2021). AD is considered a multifactorial pathology, characterized by selective neuronal death, failure in cholinergic neurotransmissions, and two main classical pathologic hallmarks, i.e., senile plaques formed by extracellular deposits of amyloid-β peptides, and intracellular neurofibrillary tangles (NFTs) of hyperphosphorylated tau protein (Serrano-Pozo et al. 2011; Guo et al. 2020; Sharma et al. 2019). The existing clinical therapies for symptomatic treatment of AD, including acetylcholinesterase (AChE) inhibitors, lack the disease-modifying potential and are associated with several side effects, such as cardiovascular and gastrointestinal adverse effects, resulting from overstimulation of peripheral cholinergic activity (Schneider 2000; Ruangritchankul et al. 2021). Therefore, there is a great need for novel multi-target therapies capable of counteracting synergic mechanisms underlying AD pathogenesis (Sharma et al. 2019; Benek et al. 2020).

Although a large research effort has been made to study amyloid-β overproduction and/or tau hyperphosphorylation, their contribution to the onset and pathogenesis of AD is still controversial. Undoubtedly, both pathological hallmarks play an important role in AD pathophysiology. Indeed, according to the classical amyloid hypothesis, Aβ aggregation triggers a cascade of events ultimately resulting in AD. However, experimental and clinical evidence, including the failure of Aβ-targeting drugs to treat AD, argues that Aβ expression may not be the primary cause of all AD, playing instead a secondary role as part of more complex processes in the CNS, including synapses degeneration, neuroinflammation, and oxidative stress (Morris et al. 2014; Kametani and Hasegawa 2018). Similarly, most of the efforts to develop tau-targeting therapies have failed in clinical trials (Du et al. 2018). More recently, a large body of evidence highlighted a strong association between AD and extensive oxidative stress (Cheignon et al. 2018; Wang et al. 2014). Interestingly, oxidative damage seems to occur in the very early stage of the disease and before the full development of the pathology, thus suggesting that oxidative imbalance might represent a central feature of AD pathogenesis (Axelsen et al. 2011; Butterfield and Boyd-Kimball 2020). Oxidative stress originates from an imbalance between the generation and detoxification of reactive oxygen species (ROS), which can play opposite roles in cells. ROS can work as signaling molecules under carefully controlled situations, modulating vital cell processes; however, especially when overproduced, ROS can react with all the major biomolecules including nucleic acid, protein, and lipids, causing cell damage and cell death eventually (Massaad 2011; Oswald et al. 2018). Since mitochondria are the main source of ROS and also a major target of oxidative damage, their dysfunction represents a prominent and early feature of AD (Wang et al. 2020; Perez Ortiz and Swerdlow 2019). Indeed, according to the “mitochondrial cascade hypothesis,” the mitochondrial dysfunction is the primary process to trigger the cascade of events that lead to sporadic AD pathogenesis (Swerdlow et al. 2014). In the light of all this, oxidative stress and the related mitochondria impairment, beyond representing essential pathological biomarkers for AD, can also serve as a promising therapeutic target (Teixeira et al. 2019; Tadokoro et al. 2020).

Recently we demonstrated that Oxotremorine-M (Oxo), a non-selective muscarinic acetylcholine receptors (mAChRs) agonist, inhibits oxidative stress and the production of pro-inflammatory cytokines in the rat hippocampal formation (Frinchi et al. 2019). Interestingly, this inhibition was exerted on baseline levels of Interleukin-1β (IL-1β), Interleukin-6 (IL-6), and oxidative stress markers, revealing a cholinergic modulatory control of neuroinflammation and oxidative stress (Frinchi et al. 2019). The reduction in IL-1β and IL-6 levels correlates with the hypothesis, proposed by Borovikova et al. (2000), according to which Acetylcholine (ACh) decrease promotes neuroinflammatory events. Oxo treatment was also able to transactivate Fibroblast growth factor receptor (FGFR) in primary hippocampal neurons, producing a significant increase in neurite outgrowth (Di Liberto et al. 2017a), and up-regulate the levels of heat shock proteins (HSPs) in the rat hippocampus (Frinchi et al. 2018). Last but not least, Oxo administration ameliorated stress-induced anxiety-like behavior by, at least in part, rescuing neurotrophic factor levels (Di Liberto et al. 2017b) and inhibiting oxidative stress and neuroinflammatory responses (Frinchi et al. 2019) in rat brain. Altogether these data point out the broad-spectrum neuroprotective and neurotrophic activity of Oxo on brain cells.

In the light of these results, taking into account the complex multifactorial pathogenesis of AD, herein we aim to explore the neuroprotective effects of Oxo treatment in differentiated SH-SY5Y cells exposed to amyloid-β peptide.

## Materials and Methods

### Aβ_1-42_ Peptide Preparation

The recombinant Aβ_1-42_ (Aβ) was produced according to the protocol described in Carrotta et al. (Carrotta et al. 2006). After production, Aβ was purified by using Ni–NTA protein purification system (Ni–NTA Fast Start Kit, ID: 30600 Qiagen), and the sample was dialyzed against PBS. After a preliminary treatment with trifluoroacetic acid (TFA), the powder of the recombinant Aβ was dissolved in 0.01 M Tris–HCl buffer, pH 7.2, and the solution was readily characterized by dynamic light scattering (DLS) at T = 15 °C (Carrotta et al. 2006).

### SH-SY5Y Culture and Differentiation

Neuroblastoma SH-SY5Y cells, generously provided by Prof. Cardile from University of Catania, were cultured in T25 tissue culture flasks in a humidified atmosphere of 95% air and 5% CO_2_ at 37 °C. Culture medium was composed of Dulbecco’s Modified Eagle’s Medium and F12 (DMEM/F12 1:1, 15-090-CV Corning), supplemented with 10% fetal bovine serum (FBS, 10270-106 ThermoFisher Scientific), penicillin/streptomycin (100 U/mL, 30-002-CI Corning) and 2 mM l-glutamine (ECB3000D EuroClone), as described in (Nuzzo et al. 2021a). Cell growth medium was changed each 3 days, and the cells were sub-cultured once they reached 85–90% confluence. The differentiation protocol was initiated the day after plating by replacing growth medium with differentiation medium containing Neurobasal medium (21103-049 ThermoFisher Scientific), 2% B27 Supplement (ThermoFisher Scientific 17504-044), penicillin/streptomycin (100 U/ml), Glutamax 1% (35050-038 ThermoFisher Scientific) plus 10 μM trans-retinoic acid (tRA, R2625 Sigma-Aldrich). Cell differentiation medium was changed each 3 days and the development of neuron-like morphology was monitored every day for up to 14 days.

### Treatment of Differentiated SH-SY5Y Cells

All treatments were performed in cells differentiated for a period of time ranging between 8 and 10 days, and all experiments were concluded at differentiation day 11.

Based on the experimental groups, the cells received the following treatment: in time-course experiments, Oxo (O100 Sigma-Aldrich) 10 µM, Nicotine (N5620 Sigma-Aldrich) 10 µM and 100 µM, for 24 h, 48 h or 72 h; in dose-effects experiments, Oxo 1 µM, 10 µM or 100 µM for 48 h; in Aβ toxicity experiments, Aβ 300 nM, 600 nM or 1200 nM for 24 h; in neuroprotection experiments against Aβ-induced neurotoxicity and oxidative stress, Aβ 600 nM for 24 h, Oxo (10 µM) + Aβ (600 nM) for 24 h (co-treatment), Nicotine (10 µM or 100 µM) + Aβ (600 nM) for 24 h (co-treatment), Oxo (10 µM) for 24 h. In some experiments cells were exposed to Atropine 100 µM (A-6883 Sigma-Aldrich) 15 min before Oxo treatment. In all the experiments the control (Ctrl) groups received the equal volume of the solvent only.

### Immunocytochemistry

Cells were grown at density of 6 × 10^4^ on glass coverslips 12 mm diameter round. Cells were fixed at different time points after differentiation (3, 7 and 10 days) with 4% formaldehyde solution for 15 min at room temperature. Cells were then washed twice with PBS 1 ×, pre-incubated in blocking solution (BSA 5 mg/mL and Triton 0.1% in PBS 1 ×) for 15 min, and incubated overnight with anti-Microtubule-associated protein 2 (MAP-2) monoclonal antibody 1:400 (M4403, Sigma-Aldrich) diluted in blocking solution. The day after, cells were washed twice with PBS 1 ×, and incubated for 1 h with a rhodamine-conjugated anti-mouse IgG Cy3 antibody 1:150 (115–165-003, Jackson ImmunoResearch). After two washing with PBS 1 ×, the coverslips were mounted on slides and examined, using the 20 × objective, under a fluorescence microscope (DMRBE, Leica Microsystems), equipped with digital video camera (Spot-RT Slider, Diagnostic Instruments, Mi, USA). The images, acquired in Tiff format, were adjusted for brightness and contrast with the camera software (SPOT Advanced software, v. 4.0.9, Diagnostic Instruments). The specificity of the primary antibody was assessed by analyzing the differential basal expression of MAP-2 protein in undifferentiated and differentiated SH-SY5Y cells (low and high, respectively), and in negative Hep G2 cells (data not shown).

### MTT Assay

Cells were grown at a density of 12 × 10^3^ cells/well on 96-wells plates in a final volume of 100 μL/well. Cell viability was assessed measuring the amount of colored formazan generated by the reduction of 3‐(4,5‐dimethylthiazol‐2‐yl)‐2,5‐diphenyltetrazolium bromide (MTT, 0.5 mg/mL, M-2128 Sigma-Aldrich) by viable cells after 3 h incubation at 37 °C. Absorbance was measured at 570 nm with background subtraction using Thermo Scientific™ Multiskan™ GO Microplate Spectrophotometer, after dissolving formazan crystals with DMSO (100 μL/well).

### Morphological Analysis

Cells were grown at density of 3 × 10^4^ on glass coverslips 12 mm diameter round. At the end of treatments, cells were fixed with 4% formaldehyde solution for 15 min at room temperature, washed twice with PBS 1x**,** and the coverslips were mounted on slides. 12 bright field images (20 ×)/coverslip were obtained using a Leica DMIL Led Inverted microscope equipped with a Leica ICC50 HD camera. The primary neurite length was measured by manually tracing the neurite from the boundary of the soma to the tip of the axon using *ImageJ 1.52v software*. All the counts were carried out in a blind manner by two independent experimenters unware of sample identity. The average value of primary neurite lengths/coverslip was employed for statistical analysis.

### Viable Cell Count

Viable cell yield was determined by Trypan Blue Exclusion Method. To this end, cells were grown at a density of 2 × 10^5^ cells/well on 24-wells plates in a final volume of 500 μL/well. After mixing 10 μL of cell suspension with 90 μL of Trypan Blue (0.4% w/v solution, T8154 Sigma-Aldrich), 10 µL of the resulting solution were pipetted in the hemocytometer chamber for viable cell counting.

### TUNEL (Terminal Deoxynucleotidyl Transferase-dUTP Nick-end Labeling) Assay

DNA fragmentation was measured on cells grown at a density of 12 × 10^3^ cells/well on 96-wells plates by the In Situ Cell Death Detection Kit, TMR red (12156792910, Roche), according to the manufacturer instructions. Briefly, after fixation, the cells were incubated with permeabilization solution for 8 min, washed with PBS, and incubated with TUNEL reaction mixture for 60 min at 37 °C in the dark. After washing with PBS, the cells were analyzed by using a fluorescent Zeiss Axio Scope 2 microscope (Carl Zeiss, Oberkochen, Germany) at a magnification of 20 ×, while fluorescence intensity was measured using the Microplate Reader GloMax fluorimeter (Promega Corporation).

### ELISA Analysis

Cells were grown at a density of 12 × 10^3^ cells/well on 96-wells plates in a final volume of 100 μL/well. The levels of IL-6 and IL-1β were determined in cell supernatants using ELISA kits (BMS213INST and BMS224INST respectively, ThermoFisher Scientific), according to the manufacturer instructions. Briefly, cell supernatant (50 µL) was added to the wells provided with the kit, already pre-coated with biotinylated monoclonal antibody to human IL-6 or IL-1β, Streptavidin-HRP and sample diluent. After 3 h of incubation, wells were washed 6 times with washing buffer, and treated with tetramethyl-benzidine substrate solution. After the addition of the Stop solution, containing 1 M Phosphoric acid, absorbance was read on a Thermo Scientific™ Multiskan™ GO Microplate Spectrophotometer using 450 nm as the primary wave length and 620 nm as the reference wave length.

### SOD Activity Levels

Total SOD enzymatic activity was measured on cells grown at a density of 12 × 10^3^ cells/well on 96-wells plates by using the SOD assay kit (19160, Sigma–Aldrich), according to manufacturer instructions. The assay exploits soluble tetrazolium salt, WST-1, that produces a water-soluble formazan dye upon reduction with a superoxide anion. SOD activity, inversely proportional to the amount of superoxide anion, was quantified by measuring the decrease in the color development at 440 nm using Thermo Scientific™ Multiskan™ GO Microplate Spectrophotometer.

### ROS Analysis

To assess ROS generation, cells were grown at a density of 12 × 10^3^ cells/well on 96-wells plates in a final volume of 100 μL/well. At the end of treatments, cells were incubated with 1 mM dichlorofluorescein diacetate (DCFH-DA, 35845 Sigma-Aldrich) for 10 min at room temperature in the dark. The conversion of non-fluorescent DCFH-DA to the highly fluorescent compound 20,70-dichlorofluorescein (DCF) by esterase activity was used to monitor the presence of peroxides due to the cellular oxidative burst. Sample fluorescence was measured by using a microplate Reader GloMax fluorimeter (Promega Corporation) at the excitation wavelength of 475 nm and emission wavelength of 555 nm. Representative pictures of ROS fluorescent signal were captured by the fluorescence Zeiss Axio Scope 2 microscope (Carl Zeiss, Oberkochen, Germany).

### Oxidation Kinetics

The oxidation kinetics was investigated in cells plated at a density of 12 × 10^3^ cells/well on 96-wells plates in a final volume of 100 μL/well. After Aβ treatment, the kinetics of ROS production was evaluated for 2 h following the addition DCFH-DA, using the Microplate Reader GloMax fluorimeter (Promega Corporation) at the excitation wavelength of 475 nm and emission wavelength 555 nm, as described in (Nuzzo et al. 2021b).

### Mitochondrial Superoxide Levels

Levels of superoxide in the mitochondria were evaluated in living cells grown at a density of 12 × 10^3^ cells/well on 96-wells plates by using MitoSOX Red mitochondrial superoxide indicator (M36008 ThermoFisher Scientific), according to manufacturer instructions. MitoSOX™ Red reagent permeates live cells where it selectively targets mitochondria: there it is rapidly oxidized by superoxide, generating a fluorescent signal upon binding to nucleic acid. Briefly, cells were incubated with MitoSOX (5 μM) for 30 min, and washed twice with PBS containing calcium and magnesium. Fluorescence was read using the Microplate Reader GloMax fluorimeter (Promega Corporation) at the excitation wavelength of 510 nm and emission wavelength 580 nm.

### Mitochondria Isolation

Cell cytosol and Mitochondria fractions were prepared using the Mitochondrial isolation kit (89874 ThermoFisher Scientific) following the manufacturer instructions. 2 × 10^7^ cells were dissolved in 200 µL of lysis buffer and centrifuged at 2000×*g* for 3 min to remove cell debris. The supernatant was centrifuged at 10,000×*g* for 5 min, the mitochondrial pellet was washed twice by centrifugation at 10,000×*g* for 10 min and resuspended in the buffer provided with the kit. After quantification by the Bradford method, an equal amount (50 μg) of mitochondrial protein was used for the subsequent analysis.

### Mitochondrial Swelling

Swelling of isolated mitochondria was evaluated by the changes in the absorbance of the mitochondrial suspensions at 540 nm using a GloMax® Discover multimode plate reader (Promega, Italy), according to the method described in (Chapa-Dubocq et al. 2018). Briefly, a volume corresponding to 50 µg of mitochondrial proteins was incubated with 50 µL of physiological buffer solution (125 mM KCl, 1 mM MgCl2, 5 mM malate, 5 mM glutamate, 1 µM EGTA, and 20 mM Tris base) at pH 7.4. The absorbance was monitored for 5 min at 37 °C at 540 nm, and the mitochondrial swelling was indicated by a decrease in the absorbance at 540 nm.

### Mitochondrial Membrane Potential Analysis

To assess mitochondrial transmembrane potential cells were grown at a density of 12 × 10^3^ cells/well on 96-wells plates in a final volume of 100 μL/well. Cells were incubated for 30 min at 37 °C with 2 mM JC-1 red dye (5,5′,6,6′-tetrachloro-1,1′,3,3′-tetraethylbenzimidazolylcarbocyanine iodide) using the MitoProbe JC-1 assay kit (M34152, ThermoFisher Scientific). When mitochondrial depolarization occurs, the dye accumulates in mitochondria, determining a fluorescence emission shift from green (∼ 529 nm) to red (∼ 590 nm). Therefore, mitochondrial depolarization was indicated by a decrease in the red/green fluorescence intensity ratio (Sivandzade et al. 2019), measured using the Microplate Reader GloMax fluorimeter (Promega Corporation).

### Statistical Analysis

An a priori sample size calculation was provided by G*Power. In details, in order to detect an effect size of f = 0.6 with 80% power in a one-way between-subjects ANOVA (four groups, alpha = 0.05), G*Power suggested 9 samples in each group.

Data analysis was performed using GraphPad Prism 8.4.3 software (GraphPad Software, Inc, La Jolla, CA, USA). Variance homogeneity was assessed by Brown-Forsythe test, while normal distribution of data was assessed by Shapiro–Wilk test. The results of the tests for normality and variance homogeneity are reported in the supplementary files. For data normally distributed, statistical evaluations were performed by one-way ANOVA, followed by Tukey Post hoc test. The relative results were presented as mean ± Standard Deviation (SD), and in some case were expressed as arbitrary units, with controls equal to 1, or as percentage of control. For data not normally distributed, statistical evaluations were performed by Kruskal–Wallis test, followed by Dunn’s multiple comparison test. The relative results were displayed as median with interquartile range and expressed as arbitrary units, with controls equal to 1. Differences in *P* value less than 0.05 were considered statistically significant.

All the statistical analyses were carried out in a blind manner by two independent experimenters unware of sample identity.

## Results

### SH-SY5Y Differentiation

**Fig. 1:**
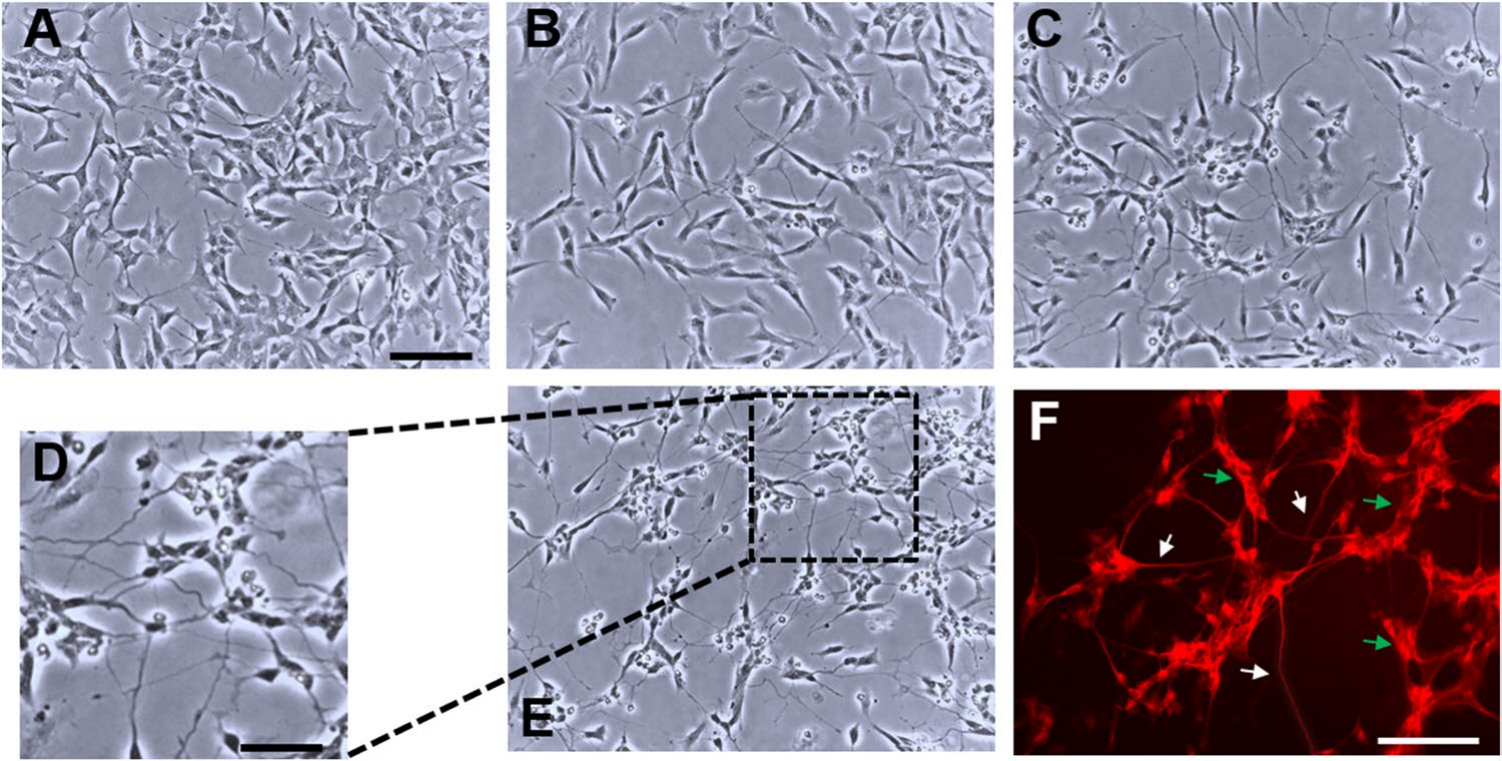
Differentiation of SH-SY5Y cells. Bright-field pictures of undifferentiated cells (**A**), cells differentiated for 3 days (**B**), 7 days (**C**) and 10 days (**E**). **D** is an enlargement of **E**. **F** MAP-2 fluorescent staining in SH-SY5Y cells differentiated for 10 days. White and green arrows indicate labeling of neurite network and cell body clusters, respectively. Scale bar: **A**, **B**, **C**, **E** 100 µm; **D** 50 µm; **F** 100 µm

The process of cell differentiation started 24 h after plating and was monitored up to 14 days. Undifferentiated cells showed a flat morphology, and a non-polarized cell body with few and short projections (Fig. 1A). During the differentiation process, cells formed extensive and elongated neuritic projections (Fig. 1B–E). Together with the development of a complex network of neurites, a distinct decrease in cell number during the differentiation process was also observed, as many cells did not survive (Fig. 1B–E).

Cells achieved a plateau of differentiation after 10 days, as confirmed by the presence of long and branched processes (Fig. 1D, E) and a full expression of MAP-2, a marker of neuronal differentiation, localized in the cell bodies and in the neurite network (Fig. 1F).

### Oxo Treatment Increases Cell Viability and Neurite Length

**Fig. 2:**
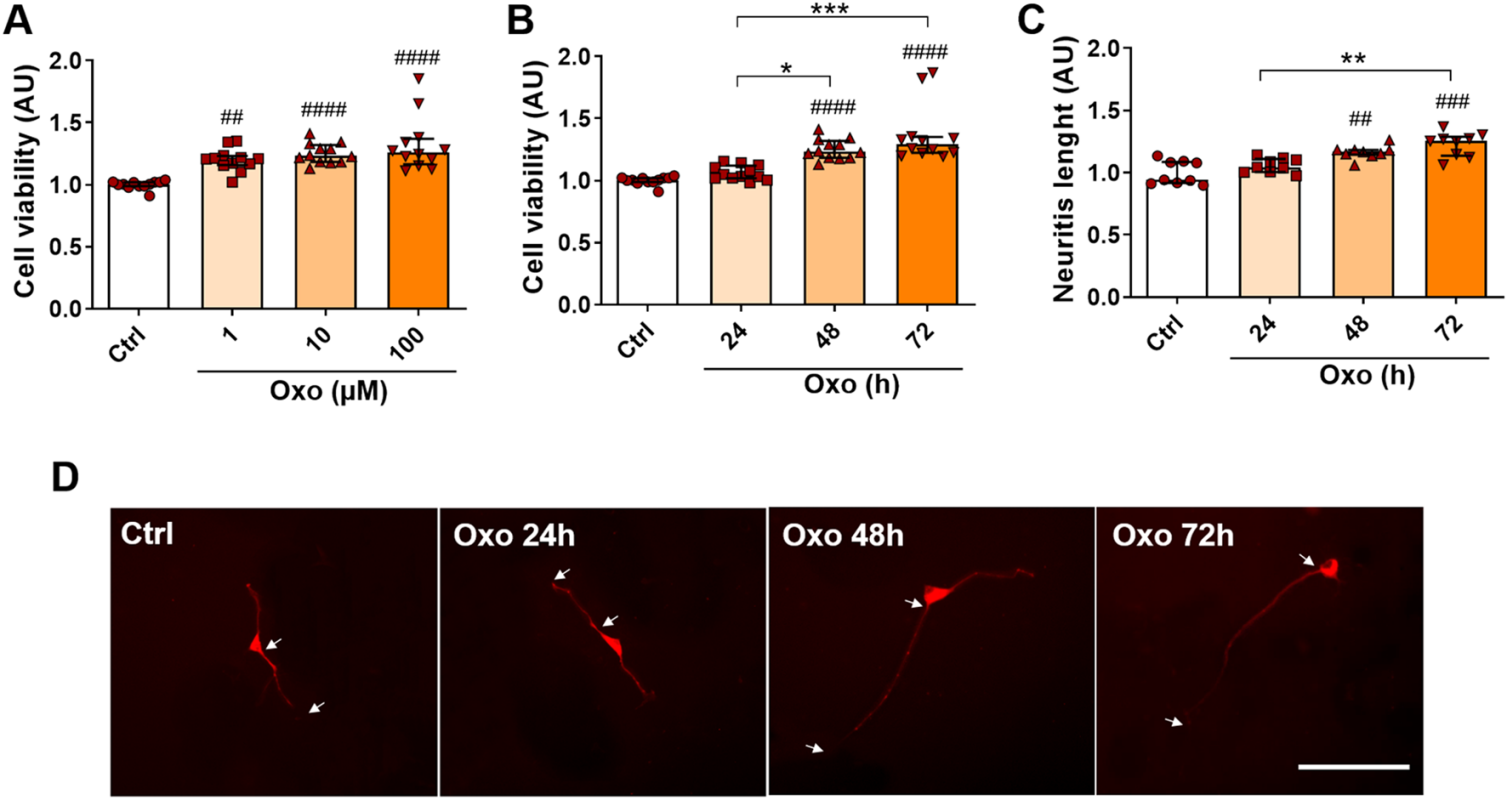
Oxo neurotrophic effects. **A** Dose–effect of Oxo treatment (48 h) on cell viability, evaluated by MTT assay. **B** Time-course of Oxo treatment (10 µM) effects on cell viability, assessed by MTT assay. **C** Time-course of Oxo treatment (10 µM) effects on neurite length. **D** Representative immunofluorescence pictures of MAP-2 staining showing the major neurite length. Arrows indicate the initial segments and terminals of neurite. Data are plotted as median and interquartile range. Scale bar 100 µm. Post hoc test: ##*P* < 0.01, ###*P* < 0.001, ####*P* < 0.0001 as compared to Ctrl group; *P < 0.05, **P < 0.01, ***P < 0.001

Dose–effect investigation of differentiated SH-SY5Y cell viability in response to Oxo exposure (48 h) were carried out. Kruskal–Wallis test (H_(47)_ = 27.64, *P* < 0.0001), followed by Dunn’s multiple comparison test, showed that Oxo treatment, at all doses, significantly increases cell viability (Fig. 2A). The above-described results were confirmed by the Trypan blue exclusion method count of viable cells (Supplementary Fig. 1A). Indeed, one-way ANOVA (F_(3,32)_ = 6.408, *P* = 0.0016), followed by Tukey’s multiple comparison test, revealed a significant dose-dependent increase in the yield of viable cells associated with 48 h Oxo 10 µM and Oxo 100 µM treatment (Supplementary Fig. 1A). Oxo 10 µM, the lowest dose exerting the greatest neurotrophic effect, was selected for all the subsequent studies. Next, time-course modulation of cell viability in response to Oxo exposure (10 µM) was investigated. Statistical evaluation of data by Kruskal–Wallis test (H _(47)_ = 37.22, *P* < 0.0001), followed by Dunn’s multiple comparison test, revealed that Oxo-induced increase in cell viability persists up to 72 h, while Oxo treatment for 24 h does not produce any significant change in cell viability (Fig. 2B). Similar results were obtained by the Trypan blue exclusion method count of viable cells, analysed by one-way ANOVA (F_(3,44)_ = 13.43, *P* < 0.0001), followed by Tukey’s multiple comparison test (Supplementary Fig. 1B). Interestingly, Oxo-induced enhancement of cell survival was completely abolished by pre-treatment with Atropine, a mAChR antagonist (Supplementary Fig. 2A), as outlined by one-way ANOVA (F_(3,44)_ = 16.68, *P* < 0.0001), followed by Tukey’s multiple comparison test. Moreover, treatment with Nicotine, at both 10 µM [Kruskal–Wallis test (H _(47)_ = 1.4116, *P* = 0.7018)] and100 µM doses [one-way ANOVA (F_(3,44)_ = 0.4976, *P* = 0.6859)], failed in inducing any significant change in cell viability (Supplementary Fig. 2B, C). Taken together, these data suggest the specific involvement of mAChRs in Oxo-induced increase of differentiated SH-SY5Y cell viability.

Since Oxo treatment was able to increase cell survival, we further tested Oxo neurotrophic effects by measuring the length of the major neurite. As shown in Fig. 2C and D, Oxo treatment (10 µM) induced a significant, time-dependent, increase in neurite length. In details, Kruskal–Wallis test (H _(35)_ = 21.50, *P* < 0.0001), followed by Dunn’s multiple comparison test, disclosed a significant increase in neurite length associated with 48 h and 72 h exposure to Oxo molecule, while Oxo treatment for 24 h failed to significantly induce primary neurite elongation (Fig. 2C, D).

### Oxo Treatment Reduces Cell Death, DNA Fragmentation, and Neurite Atrophy Induced by Aβ Treatment

**Fig. 3:**
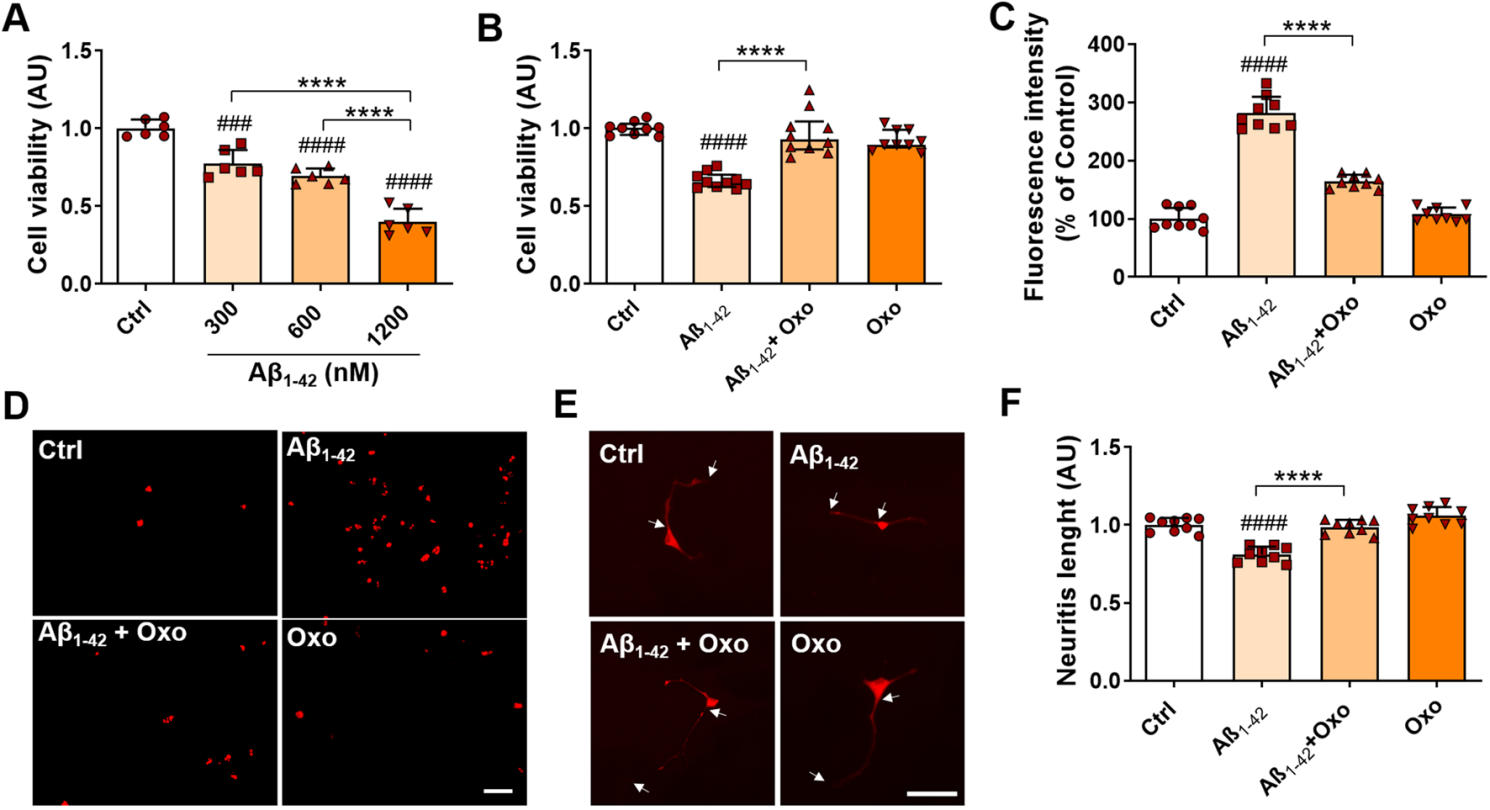
Neuroprotective and neurotrophic role of Oxo treatment against Aβ_1-42_-induced cell damage. **A** Dose–effect of Aβ_1-42_ treatment (24 h) on SH-SY5Y cell viability, evaluated by MTT assay. **B** Quantification of cell viability by MTT test in Ctrl cells, cells treated with Aβ1-42 (600 nM, 24 h), Aβ_1-42_ (600 nM, 24 h) + Oxo (10 µM, 24 h), and Oxo alone (10 µM, 24 h). **C** TUNEL fluorescence intensity quantification. **D** Representative picture of TUNEL-positive cells, visualized by red fluorescent labeling. **E** Representative immunofluorescence pictures of MAP-2 staining showing major neurite length. Arrows indicate initial segments and terminals of neurite. **F** Quantification of the major neurite length. Data are plotted as mean and SD in (**A**), (**C**) and (**F**), and as median and interquartile range in (**B**). Scale bar 50 µm. Post hoc test: ###*P* < 0.001, ####*P* < 0.0001 as compared to Ctrl group; ****P < 0.0001

We next assessed the neuroprotective effect of Oxo treatment in an in vitro model of AD induced by cell exposure to Aβ peptide. Cells treated for 24 h with Aβ showed a dose-dependent significant decrease in cell viability, as assessed by MTT test (Fig. 3A). In details, one-way ANOVA (F_(3,20)_ = 72.65, *P* < 0.0001), followed by Tukey’s multiple comparison test, revealed significant decrease of cell viability for all the Aβ tested doses, with the most significant effects associated with Aβ 600 nM and Aβ 1200 nM treatment. Aβ 600 nM dose was chosen for the subsequent investigations aimed to assess neuroprotective effects of Oxo treatment. Statistical evaluation of cell viability data by Dunn’s post hoc test following Kruskal–Wallis test highlighted that Oxo treatment (10 µM, 24 h), which per se does not induce any significant change in cell survival, fully restores cell viability impaired by Aβ treatment (H_(37)_ = 24.25, *P* < 0.0001) (Fig. 3B). Intriguingly, pre-treatment with Atropine significantly inhibited Oxo neuroprotective effects, as shown by one-way ANOVA (F_(3,39)_ = 14.76, *P* < 0.0001), followed by Tukey’s multiple comparison test (Supplementary Fig. 3A), while Nicotine treatment was not able to preserve cell viability impaired by Aβ treatment, as outlined by Dunn’s post hoc test following Kruskal–Wallis test (H_(41)_ = 18.08, *P* = 0.0004) (Supplementary 3B).

The neuroprotective effect of Oxo treatment against Aβ-induced cell death was further tested by TUNEL assay. The relative results, shown in Fig. 3C and D, and analyzed by one-way ANOVA (F_(3,32)_ = 182.2, *P* < 0.0001) followed by Tukey’s multiple comparisons test, demonstrated that Oxo treatment (10 µM, 24 h) significantly reduces DNA fragmentation induced by Aβ treatment.

Since neuritic abnormalities, caused by Aβ-induced interference with tubulin assembly, represent a major hallmark of AD pathology (Petratos et al. 2008), we next investigated the effect of Oxo treatment in modulating neurite length in Aβ-exposed cells. Statistical evaluation of data by one-way ANOVA (F_(3,32)_ = 41.15, *P* < 0.0001) followed by Tukey’s multiple comparisons test proved that Oxo treatment (10 µM, 24 h), which per se does induce any significant change in neurite length, significantly counteracts the decrease in neurite length induced by Aβ treatment (Fig. 3E, F).

### Oxo Treatment Counteracts Oxidative Stress Induced by Aβ Treatment

The strict association between AD pathogenesis and the generation of oxidative stress (Cheignon et al. 2018), prompted us to investigate the impact of Oxo treatment on the modulation of Aβ-induced oxidative stress-related parameters, including ROS production and SOD activity.

**Fig. 4:**
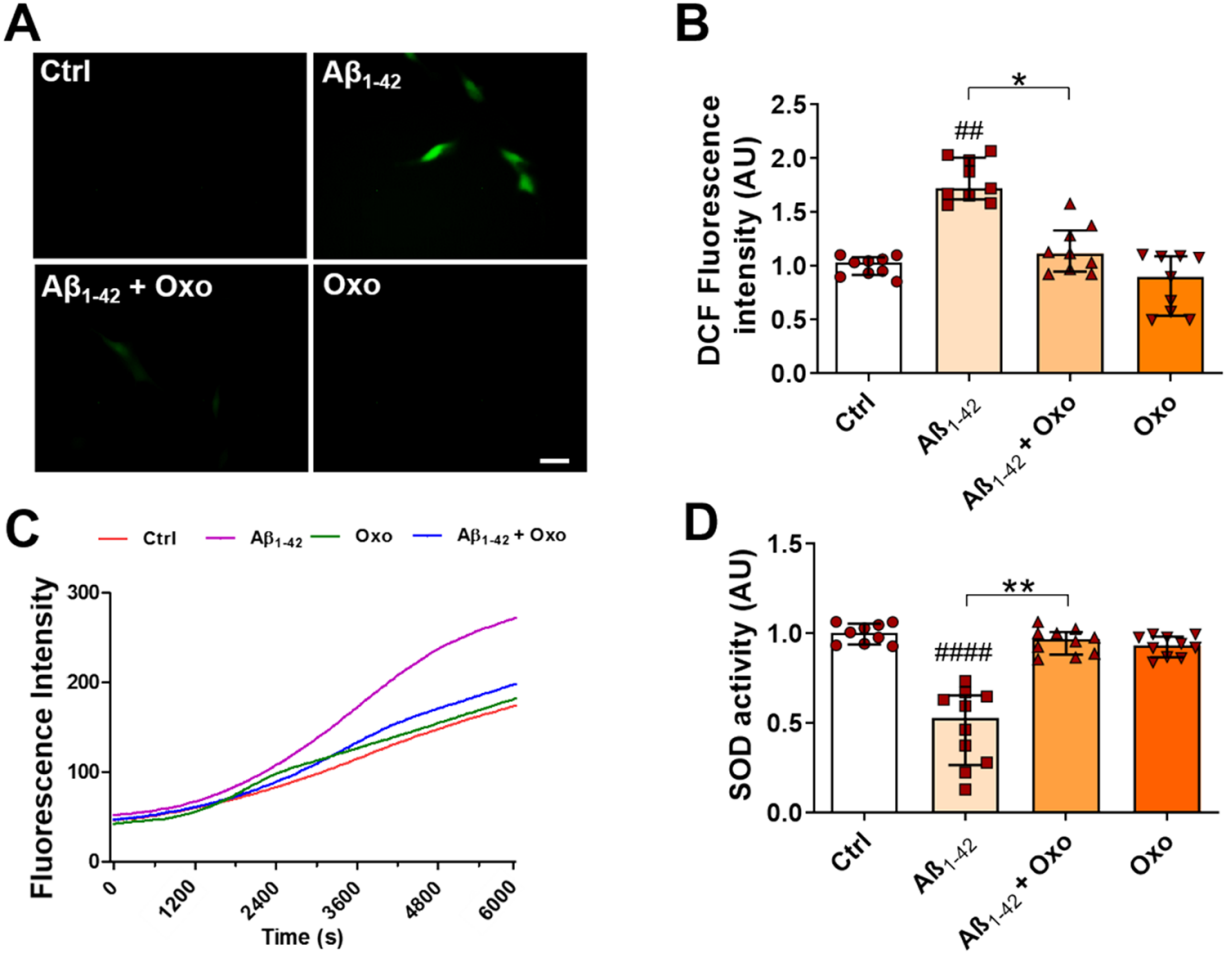
Antioxidant effects of Oxo treatment against Aβ_1-42_-induced oxidative stress. **A** Representative picture of ROS generation, visualized by green fluorescent DCF signal, in Ctrl cells, cells treated with Aβ_1-42_ (600 nM, 24 h), Aβ_1-42_ (600 nM, 24 h) + Oxo (10 µM, 24 h), and Oxo alone (10 µM, 24 h). **B** DCF fluorescence intensity quantification. **C** Kinetics of ROS generation monitored using DCFH-DA fluorescence assay. **D** Quantification of SOD activity. Data are plotted as median and interquartile range. Scale bar 50 µm. Post hoc test: ##*P* < 0.01, ####*P* < 0.0001 as compared to Ctrl group, *P < 0.5, **P < 0.01

Fluorescence microscope inspection of DCFH-DA signal intensity (Fig. 4A) and its quantification (Fig. 4B) pointed out the Oxo modulation of ROS production. In details, Dunn’s post hoc test following Kruskal–Wallis test revealed that Oxo treatment (10 µM, 24 h) completely elapses ROS increase driven by Aβ exposure (H_(35)_ = 22.35, *P* < 0.0001).

The kinetics of ROS production after exposure of SH-SY5Y cells to Aβ showed a rapid acceleration (purple curve in Fig. 4C), whereas concomitant treatment with Oxo was effective in lowering and delaying ROS generation (blue curve in Fig. 4C).

Besides the capability of decreasing the amount of intracellular ROS, Oxo treatment (10 µM, 24 h) was also able to significantly counteract the decrease in SOD activity induced by cell exposure to Aβ (Fig. 4D), as outlined by Dunn’s post hoc test following Kruskal–Wallis test (H_(38)_ = 24.91, *P* < 0.0001).

### Oxo Treatment Blocks Mitochondrial Oxidative Stress and Functionality Impairment Induced by Aβ Treatment

**Fig. 5:**
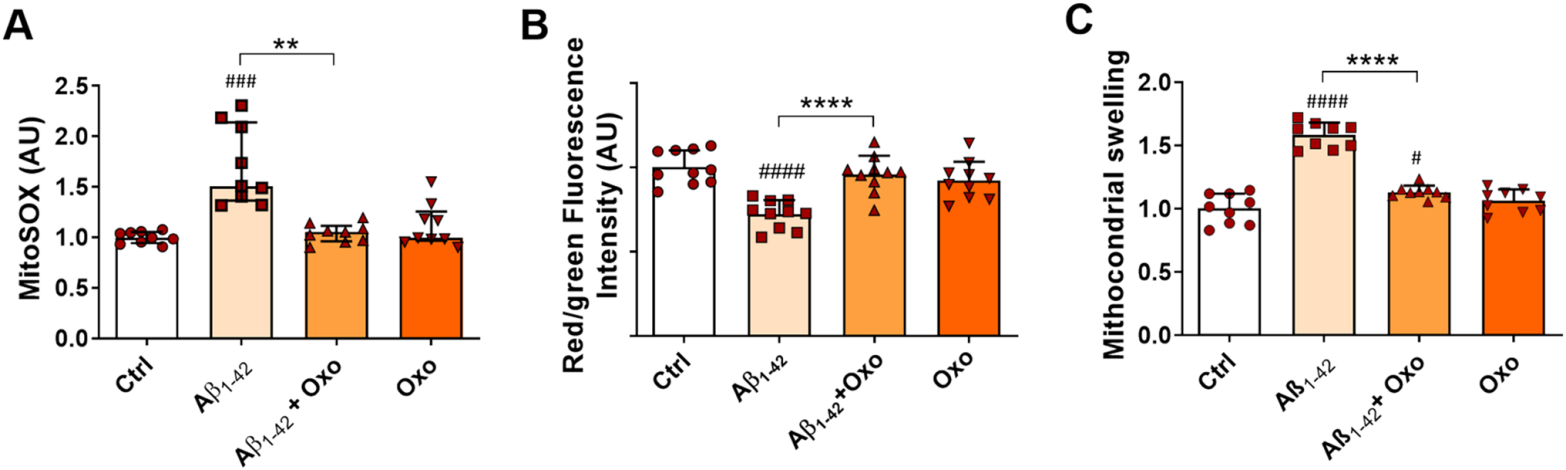
Mitoprotective role of Oxo treatment against Aβ_1-42_-induced mitochondria impairment. **A** Quantification of mitochondrial superoxide by MitoSOX Red mitochondrial superoxide indicator in Ctrl cells, cells treated with Aβ_1-42_ (600 nM, 24 h), Aβ_1-42_ (600 nM, 24 h) + Oxo (10 µM, 24 h), and Oxo alone (10 µM, 24 h). **B** Measurement of mitochondrial membrane potential by quantification of JC-1 red/green fluorescence intensity ratio. **C** Measurement of mitochondria swelling by quantification of mitochondria absorbance. Data are plotted as median and interquartile range in (**A**), and as mean and SD in (**B**) and (**C**). Post hoc test: ###*P* < 0.001, ####*P* < 0.0001 as compared to Ctrl group; **P < 0.01, ****P < 0.0001

Exacerbated ROS production is bi-directionally associated with mitochondria oxidative damage (Wang et al. 2014). In order to assess the impact of Oxo treatment on Aβ-induced mitochondrial oxidative stress, we first evaluated signal intensity produced by cell incubation with MitoSOX, a fluorogenic dye specifically targeted to mitochondria, which produces red fluorescence once oxidized by mitochondrial superoxide. As shown in Fig. 5A and by Dunn’s post hoc test following Kruskal–Wallis test (H_(35)_ = 18.36, *P* = 0.004), Oxo treatment (10 µM, 24 h) was able to significantly counteract the increase in MitoSOX red signal induced by cell exposure to Aβ.

Mitochondrial oxidative stress causes the impairment of mitochondria functionality and morphology, triggering the opening of mitochondrial permeability transition pore (MPTP) which leads to the collapse of the mitochondrial membrane potential, increased permeability, and subsequent osmotic swelling of the organelles (Picone et al. 2014).

Variations in the physiological mitochondrial membrane potential were measured as changes in the accumulation of JC-1 cyanine dye (Sivandzade et al. 2019). Evaluation of data by one-way ANOVA (F_(3,36)_ = 14.85, *P* < 0.0001) followed by Tukey’s multiple comparisons test underlined a reduction in the red to green fluorescence intensity ratio, indicative of mitochondrial depolarization, associated with cell exposure to Aβ, while concomitant treatment with Oxo significantly blocked this effect (Fig. 5B).

Finally, changes in mitochondrial morphology were analyzed by measuring the absorbance of isolated mitochondria. Mitochondria swelling was significantly increased following cell exposure to Aβ, while co-treatment with Oxo significantly counteracted this effect (Fig. 5C), as statistically assessed by one-way ANOVA (F_(3,32)_ = 72.75, *P* < 0.0001) followed by Tukey’s multiple comparisons test.

### Oxo Treatment and Aβ Exposure Do Not Modulate the Release of Pro-inflammatory Cytokines

**Fig. 6:**
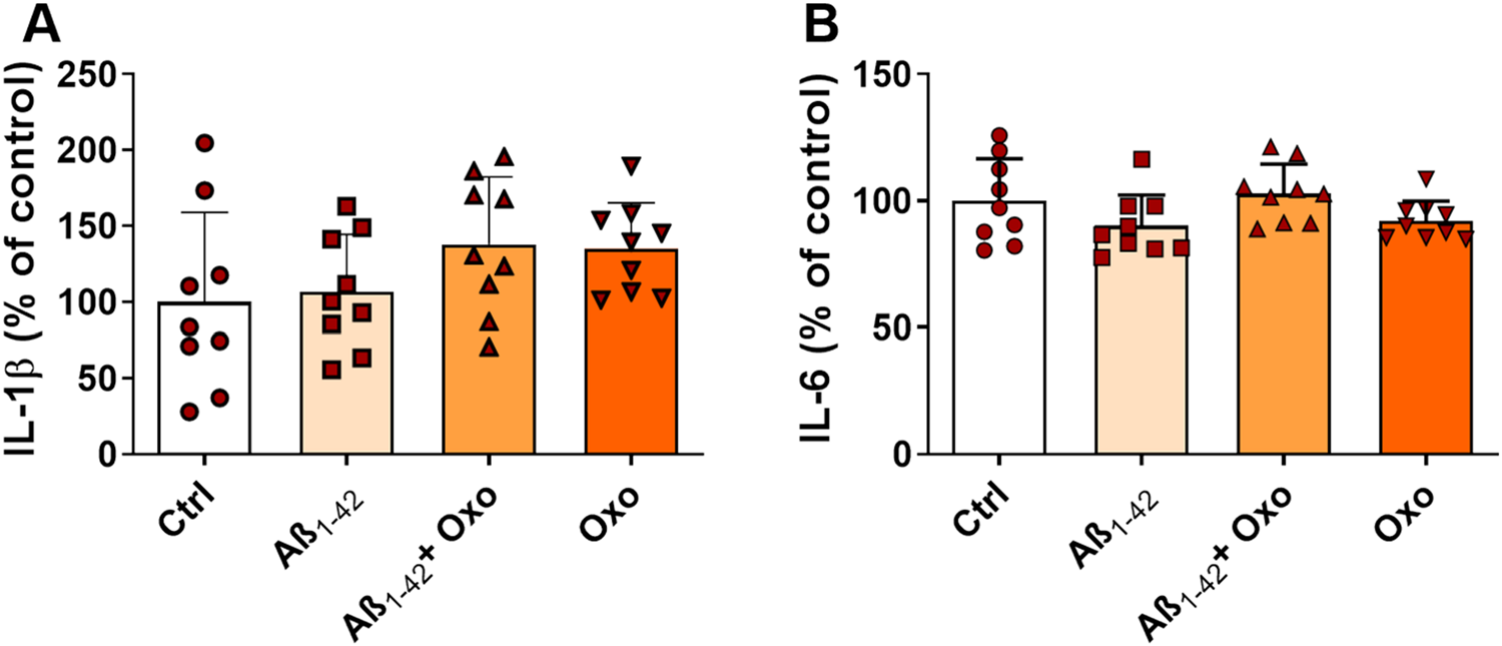
Modulation of ILs levels by Oxo treatment. **A** Elisa quantification of IL-1β levels in Ctrl cells, cells treated with Aβ_1-42_ (600 nM, 24 h), Aβ_1-42_ (600 nM, 24 h) + Oxo (10 µM, 24 h), and Oxo alone (10 µM, 24 h). **B** Elisa quantification of IL-6 levels. Data are plotted as mean and SD

A large body of evidence suggests that AD pathogenesis is linked to chronic neuroinflammation response, characterized by the release of inflammatory mediators, that further worsen the deposition of Aβ, oxidative stress, neuronal dysfunctions, and death (Picca et al. 2020). In order to assess the impact of Oxo treatment on pro-inflammatory cytokines release and neuroinflammation modulation, we measured the amount of IL-1β and IL-6 secretion following cell exposure to either Aβ or Oxo. The results, shown in Fig. 6, revealed that neither Aβ exposure nor Oxo treatment were able to significantly modify basal IL-1β and IL-6 secretion, as also confirmed by the outcome of one-way ANOVA analysis of data (IL-1β: F_(3,32)_ = 1.756, *P* = 0.1754: IL-6: F_(3,32)_ = 2.187, *P* = 0.1088, respectively).

## Discussion

In this study, we have demonstrated that treatment with Oxo, a non-selective mAChRs agonist, exerts neurotrophic and neuroprotective effects in SH-SY5Y differentiated cells submitted to Aβ-induced neurotoxic damage, by modulating oxidative stress response and mitochondria functionality.

mAChRs family includes five G-protein coupled receptor subtypes (M1-M5) widely distributed throughout the brain, whose activation has been associated with the regulation of different brain functions and cellular processes, such as synaptic plasticity, neural stem cell proliferation, neuronal differentiation, and survival (Resende and Adhikari 2009; Brown 2010; Picciotto et al. 2012; Di Liberto et al. 2014; Giordano et al. 2009). In this context, we have previously shown that Oxo is able to (i) transactivate FGFR and produce a significant increase in neurite outgrowth in primary hippocampal neurons (Di Liberto et al. 2017a); (ii) ameliorate stress-induced anxiety-like behavior and rescue neurotrophic factor levels in the rat brain (Di Liberto et al. 2017a); (iii) up-regulate HSPs expression in the rat hippocampus (Frinchi et al. 2018); and (iv) modulate neuroinflammation and oxidative stress in the same brain area (Frinchi et al. 2019).

Here we first investigated Oxo neurotrophic effects in SH-SY5Y differentiated cells. The neuroblastoma cells were differentiated into a mature cholinergic neuron phenotype by the use of RA, which is known to induce an increase in the expression of ACh transferase and vesicular monoamine transporter (Lopes et al. 2010; Presgraves et al. 2004; de Medeiros et al. 2019). By checking the changes in cell morphology and in MAP-2 expression, we found that cells were functionally mature after 10 days of differentiation, showing a decrease in the proliferation rate and a full expression of MAP-2 protein, associated with the development of a complex network of elongated neuritic projections. At this stage, Oxo treatment induced a dose- and time-dependent increase in neurite outgrowth and cell viability, probably by inhibiting cell death associated with the process of differentiation (Shipley et al. 2016). In line with our data, other studies reported that activation of the M1 muscarinic receptor subtype in rat pheochromocytoma PC12 cells stably expressing cloned M1 receptor with Oxo stimulates outgrowth of neurite‐like processes (Pinkas-Kramarski et al. 1992), growth arrest, and inhibition of apoptotic death induced by serum deprivation (Lindenboim et al. 1995).

Interestingly, it has been demonstrated that Oxo can also activate nicotinic ACh receptors (nAChRs) (Reitstetter et al. 1994; Akk and Auerbach 1999), suggesting that some of the effects observed in this study might be dependent, at least in part, on ACh ionotropic receptors. Indeed, nAChR activation promotes neurotrophic signaling in brain and neuronal cells, including the positive modulation of neurogenesis, synaptic plasticity (Belluardo et al. 2008; Mudò et al. 2007b), and neuroprotection (Mudò et al. 2007a). Here we found that Oxo-induced increase in cell survival was abolished by the co-treatment with the muscarinic antagonist Atropine. In addition, treatment with Nicotine failed in increasing cell viability, clearly suggesting the specific involvement of mAChRs in Oxo-induced enhancement of differentiated SH-SY5Y cell survival.

Since mAChR activation induces the activation of several biochemical cascades (Deguil et al. 2008), we can speculate that different synergic processes may contribute to Oxo neurotrophic functions, such as the transactivation of neurotrophic factor receptors (Di Liberto et al. 2014, 2017a, 2019), and the up-regulation of neurotrophic factor and HSP expression (Di Liberto et al. 2017a; Frinchi et al. 2018).

AD pathogenesis and progression are strictly linked to a complicated interplay between many contributor mechanisms, including abnormal protein aggregates, cholinergic neurodegeneration, oxidative stress, neuroinflammation, and altered metal homeostasis (Liu et al. 2019). Cholinergic neurodegeneration is considered a critical pathological change that correlates with cognitive impairment in AD, and drugs inhibiting AChE activity currently represent the most available clinical symptomatic treatment for AD patients (Du et al. 2018). However, AChE inhibitors show a limited therapeutic potential, and long-term treatment offers no disease-modifying effects and severe gastrointestinal/cardiovascular side reactions (Sharma et al. 2019), thus suggesting the need for novel multi-target drugs to achieve a therapeutic synergy in AD (Benek et al. 2020; Savelieff et al. 2019).

An increasing body of evidence argues that mAChR stimulation may exert beneficial effects in AD. Indeed M1 receptors, in addition to their role in the modulation of cholinergic neurotransmission, have the potential to regulate the processing of pathological amyloid and tau, thus alleviating the major hallmarks of AD and extending the lifespan of terminally sick mice (Scarpa et al. 2020). Interestingly, a recent study suggested that cholinergic neurons in the basal forebrain can control inflammation and innate immune responses in mice, pointing out that a disruption of this mechanism in AD could exacerbate the pathology (Lehner et al. 2019). Here we show that Oxo treatment protects differentiated SH-SY5Y cells from Aβ-induced neurotoxic effects by preserving cell viability, DNA structure, and neurite length. Our data are further supported by other investigations showing that mAChRs stimulation blocks β-Amyloid fragment 31-35-induced apoptosis in cultured cortical neurons (Yan et al. 2000), and decreases Aβ levels and tau hyperphosphorylation (Fisher et al. 2003; Fisher 2007).

It has been largely demonstrated that nAChRs, especially the α7 subtype, can be used as therapeutic target for AD. Aβ can interact with nAChRs, exerting an inhibitory or excitatory effect depending on the system used and on Aβ concentration (Lombardo and Maskos 2015). Furthermore, activation of nAChRs leads to protective effect against Aβ-exerted toxicity by multiple mechanisms, including the stimulation of the cholinergic pathway, the modulation of inflammation, and the clearance of Aβ (Lombardo and Maskos 2015; Roberts et al. 2021; Takata et al. 2022). However, clinical trials exploring the beneficial effects of nAChR activation in AD patients have been so far complicated by adverse effects or little improvement (Hoskin et al. 2019). Here we found that Nicotine treatment is not able to inhibit Aβ-induced degeneration of differentiated SH-SY5Y cells, while Atropine treatment fully abolished Oxo recovery of cell survival. Although our data clearly indicate the specific involvement of mAChRs in Oxo protective effects, the involvement of nAChR activation in a more physiological context, including primary neurons or brain tissue, cannot be excluded at this stage.

Oxidative stress is a condition of cell insult generated by the imbalance between ROS production and cellular antioxidant capacity, due to enhanced ROS generation and/or dysfunction of the antioxidant system. ROS, generated in a low amount in normal conditions, act as essential signaling molecules, regulating important neuronal functions such as synaptic plasticity and neuronal polarity (Massaad 2011; Oswald et al. 2018). However, when the delicate balance between the rate of ROS production and the rate of their clearance by antioxidants is impaired, ROS oxidize all major biomolecules, leading to neuronal damage and subsequent death. Interestingly, oxidative damage seems to occur in the very early stage of AD and prior to the full development of the pathology, thus suggesting that oxidative imbalance might represent a central feature of its pathogenesis (Barnham et al. 2004). Here we found that Oxo treatment reduces ROS levels in Aβ-treated SH-SY5Y differentiated cells, thus revealing a new mechanism contributing to Oxo neuroprotective properties against Aβ-induced neurotoxicity. These results support previous findings reporting the ability of Oxo treatment to significantly decrease ROS levels in the rat hippocampus (Frinchi et al. 2019), and are corroborated by the observation that scopolamine (a mAchRs antagonist) treatment or M_1_ receptor deficiency produces an increase in oxidative stress (Balaban et al. 2017; Wong-Guerra et al. 2017; Laspas et al. 2019). On the contrary Xanomeline, a mAchR agonist, effective in attenuating behavioral disturbances in AD patients, protects cortical cells from oxygen–glucose deprivation via inhibiting oxidative stress and apoptosis (Xin et al. 2020).

Inactivation and deficiency of antioxidant enzymes reduce the clearance of free radicals and generate a state of oxidative stress in cells (Uttara et al. 2009). SODs are metal-containing proteins that catalyze the removal of superoxide, generating water peroxide as a final product of the dismutation (Limon-Pacheco and Gonsebatt 2009). SOD activity reduction and the subsequent ROS accumulation are well documented in AD brain (Wojsiat et al. 2018; Marcus et al. 1998). Here we demonstrated that Oxo treatment rescues SOD activity, impaired by cell exposure to Aβ, in agreement with data showing the mAchR modulation of SOD activity in the rat hippocampus (Frinchi et al. 2018) and in cultured rat cardiomyoblasts (Sun et al. 2014).

According to the “mitochondrial cascade hypothesis,” the mitochondrial dysfunction and the related vicious downward spiral with ROS production, are prominent and early features of AD, and represent the primary process to trigger the cascade of events that lead to AD pathogenesis (Cenini et al. 2019; Wang et al. 2020). Indeed, almost all aspects of mitochondrial function have been reported to be impaired in AD, including mitochondrial morphology, number and biogenesis, oxidative phosphorylation, mitochondrial membrane potential, Ca^2+^ buffering, and mitophagy (Wang et al. 2020; Perez Ortiz and Swerdlow 2019). Hence, maintenance of mitochondrial functions may represent a novel promising therapeutic strategy for AD (Wang et al. 2020). In this study we found that Oxo treatment specifically counteracts Aβ-induced mitochondrial oxidative stress and the related alteration in mitochondrial membrane potential and mitochondrial swelling. In agreement with our data, it has been shown that activation of M1 receptors is neuroprotective against glutamate-induced apoptosis in retinal neurons by maintaining calcium homeostasis and mitochondrial membrane potential (Zhou et al. 2008), while scopolamine treatment increases intracellular calcium level leading to increase in oxidative stress and mitochondrial dysfunction in the hippocampus (Balaban et al. 2017; Garabadu and Sharma 2019).

A growing body of experimental evidence suggests that AD pathogenesis is not restricted to neuronal cells, but strongly involves immunological mechanisms in the brain. Indeed, misfolded and aggregated proteins activate an innate immune response, primarily involving microglia and astrocytes, characterized by the release of inflammatory mediators, that worsen the deposition of Aβ and lead to dysfunctions in neurons, playing a key role in disease pathogenesis, progression, and severity (Heneka et al. 2015; Leng and Edison 2021; Picca et al. 2020). Although it has been previously shown that Oxo exerts anti-inflammatory effects in the rat hippocampus (Frinchi et al. 2019), here we did not detect any variation in IL-1β and IL-6 secretion following cell exposure to either Aβ or Oxo. The low levels of cytokines detected in the cell medium of differentiated SH-SY5Y cells, coupled with cell unresponsiveness to both Aβ and Oxo treatment, clearly suggest that neuroinflammation is a process mainly mediated by glial cells. Similar results were also obtained when SH-SY5Y cells were exposed to a combination of lipopolysaccharide (LPS) plus interferon-γ (IFN-γ), or Aβ peptide 1–40, whereas a release of IL-1β and IL-6 was observed in human monocytic and astrocytic cells under the same treatment (Klegeris and McGeer 2001). However, other investigations showed that both differentiated and undifferentiated SH-SY5Y cells release pro-inflammatory cytokines when exposed to different inflammatory agents, including LPS (Pandur et al. 2018) and cholesterol metabolite (Ma et al. 2019), suggesting that the differentiation protocol and the inflammation model may strongly affect cell response.

In conclusion, AD is a complex and multifactorial neurodegenerative disorder, characterized by the deposition of pathological proteins, impaired neurotransmission, extensive oxidative stress, mitochondrial dysfunctions, and neuroinflammation. Accordingly, therapeutics targeting only one of these AD-related pathological hallmarks have not yet been successful as disease-modifying treatment, thus suggesting the need for multi-target drugs to achieve a therapeutic synergy in AD. Here we demonstrate for the first time that Oxo, a non-selective mAChRs agonist, exerts neurotrophic effects in neuronal-like cells, and protects against Aβ-induced neurotoxic damage by enhancing cell survival, neurite growth, the overall response against oxidative stress, and mitochondria functionality. In the light of the present results, preclinical studies employing in vivo models of AD aimed to explore Oxo therapeutic potential are urgently needed.

## Supplementary Information

Below is the link to the electronic supplementary material.

Supplementary file1 (DOCX 460 KB)

## Data Availability

The datasets generated during and/or analysed during the current study are available from the corresponding author on reasonable request.
