# Supplementary material for: Neuroprotective and Antioxidant Role of Oxotremorine-M, a Non-selective Muscarinic Acetylcholine Receptors Agonist, in a Cellular Model of Alzheimer Disease
Source: Cell Mol Neurobiol. 2022 Sep 3;43(5):1941–56. doi: 10.1007/s10571-022-01274-9 (PMC10287780; doi:10.1007/s10571-022-01274-9)
Supplement: Supplementary file 1 — Supplementary file1 (DOCX 460 KB) [file 10571_2022_1274_MOESM1_ESM.docx]

Supplementary information- Cellular and Molecular Neurobiology

**Neuroprotective and antioxidant role of Oxotremorine-M, a non-selective muscarinic acetylcholine receptors agonist, in a cellular model of Alzheimer disease**

Domenico Nuzzo, Monica Frinchi, Costanza Giardina, Miriana Scordino, Mariachiara Zuccarini, Chiara De Simone, Marta Di Carlo, Natale Belluardo, Giuseppa Mudò^*^, Valentina Di Liberto^*^

^*^Corresponding authors: Università di Palermo, Dipartimento di Biomedicina, Neuroscienze e Diagnostica Avanzata, corso Tukory 129, 90134, Palermo, Italy. [giuseppa.mudo@unipa.it](mailto:giuseppa.mudo@unipa.it); [valentina.diliberto@unipa.it](mailto:valentina.diliberto@unipa.it)

GraphPad Prism 8.4.3 software (GraphPad Software, Inc, La Jolla, CA, USA) was employed to assess variance homogeneity (Brown-Forsythe test) and normal distribution of data (Shapiro-Wilk test). The extracted results are reported below.

Figure 2A

| **Brown-Forsythe test** |  |
| --- | --- |
| F (DFn, DFd) | 3,242 (3, 44) |
| P value | 0,0309 |
| P value summary | * |
| Are SDs significantly different (P < 0.05)? | Yes |
|  |  |
| \| **Shapiro-Wilk test** \| **Ctrl** \| **Oxo 24h** \| **Oxo 48h** \| **Oxo 72h** \| \| --- \| --- \| --- \| --- \| --- \| \| W \| 0,8190 \| 0,9370 \| 0,9605 \| 0,8132 \| \| P value \| 0,0155 \| 0,4601 \| 0,7914 \| 0,0133 \| \| Passed normality test (alpha=0.05)? \| No \| Yes \| Yes \| No \| \| P value summary \| * \| ns \| ns \| * \| |  |

Figure 2B

| **Brown-Forsythe test** |  |
| --- | --- |
| F (DFn, DFd) | 2,984 (3, 44) |
| P value | 0,0413 |
| P value summary | * |
| Are SDs significantly different (P < 0.05)? | Yes |

| **Shapiro-Wilk test** | **Ctrl** | **Oxo 1 µM** | **Oxo 10 µM** | **Oxo 100 µM** |
| --- | --- | --- | --- | --- |
| W | 0,8190 | 0,9300 | 0,9605 | 0,6860 |
| P value | 0,0155 | 0,3798 | 0,7914 | 0,0006 |
| Passed normality test (alpha=0.05)? | No | Yes | Yes | No |
| P value summary | * | ns | ns | *** |

|  |  |  |  |  |
| --- | --- | --- | --- | --- |

Figure 2C

| **Brown-Forsythe test** |  |
| --- | --- |
| F (DFn, DFd) | 1,283 (3, 32) |
| P value | 0,2970 |
| P value summary | ns |
| Are SDs significantly different (P < 0.05)? | No |

| **Shapiro-Wilk test** | **Ctrl** | **Oxo 24h** | **Oxo 48h** | **Oxo 72h** |
| --- | --- | --- | --- | --- |
| W | 0,8286 | 0,9549 | 0,9109 | 0,9392 |
| P value | 0,0431 | 0,7442 | 0,3226 | 0,5736 |
| Passed normality test (alpha=0.05)? | No | Yes | Yes | Yes |
| P value summary | * | ns | ns | ns |

Figure 3A

| **Brown-Forsythe test** |  |
| --- | --- |
| F (DFn, DFd) | 0,3569 (3, 20) |
| P value | 0,7847 |
| P value summary | ns |
| Are SDs significantly different (P < 0.05)? | No |

| **Shapiro-Wilk test** | **Ctrl** | **Aβ 300 nM** | **Aβ 600 nM** | **Aβ 1200 nM** |
| --- | --- | --- | --- | --- |
| W | 0,8606 | 0,8600 | 0,8967 | 0,8846 |
| P value | 0,1913 | 0,1891 | 0,3546 | 0,2909 |
| Passed normality test (alpha=0.05)? | Yes | Yes | Yes | Yes |
| P value summary | ns | ns | ns | ns |

Figure 3B

| **Brown-Forsythe test** |  |
| --- | --- |
| F (DFn, DFd) | 3,189 (3, 34) |
| P value | 0,0359 |
| P value summary | * |
| Are SDs significantly different (P < 0.05)? | Yes |

| **Shapiro-Wilk test** | **Ctrl** | **Aβ** | **Aβ + Oxo** | **Oxo** |
| --- | --- | --- | --- | --- |
| W | 0,9432 | 0,9209 | 0,9039 | 0,9084 |
| P value | 0,6163 | 0,3642 | 0,2419 | 0,3048 |
| Passed normality test (alpha=0.05)? | Yes | Yes | Yes | Yes |
| P value summary | ns | ns | ns | ns |

Figure 3C

| **Brown-Forsythe test** |  |
| --- | --- |
| F (DFn, DFd) | 1,870 (3, 32) |
| P value | 0,1546 |
| P value summary | ns |
| Are SDs significantly different (P < 0.05)? | No |

| **Shapiro-Wilk test** | **Ctrl** | **Aβ** | **Aβ + Oxo** | **Oxo** |
| --- | --- | --- | --- | --- |
| W | 0,8487 | 0,8920 | 0,9334 | 0,8428 |
| P value | 0,0722 | 0,2091 | 0,5146 | 0,0620 |
| Passed normality test (alpha=0.05)? | Yes | Yes | Yes | Yes |
| P value summary | ns | ns | ns | ns |

Figure 3F

| **Brown-Forsythe test** |  |
| --- | --- |
| F (DFn, DFd) | 0,2026 (3, 32) |
| P value | 0,8939 |
| P value summary | ns |
| Are SDs significantly different (P < 0.05)? | No |

| **Shapiro-Wilk test** | **Ctrl** | **Aβ** | **Aβ + Oxo** | **Oxo** |
| --- | --- | --- | --- | --- |
| W | 0,8841 | 0,9216 | 0,8841 | 0,9549 |
| P value | 0,1733 | 0,4055 | 0,1733 | 0,7442 |
| Passed normality test (alpha=0.05)? | Yes | Yes | Yes | Yes |
| P value summary | ns | ns | ns | ns |

Figure 4B

| **Brown-Forsythe test** |  |
| --- | --- |
| F (DFn, DFd) | 2,937 (3, 32) |
| P value | 0,0481 |
| P value summary | * |
| Are SDs significantly different (P < 0.05)? | Yes |

| **Shapiro-Wilk test** | **Ctrl** | **Aβ** | **Aβ + Oxo** | **Oxo** |
| --- | --- | --- | --- | --- |
| W | 0,9182 | 0,8900 | 0,9038 | 0,8128 |
| P value | 0,3776 | 0,1997 | 0,2747 | 0,0286 |
| Passed normality test (alpha=0.05)? | Yes | Yes | Yes | No |
| P value summary | ns | ns | ns | * |

Figure 4D

| **Brown-Forsythe test** |  |
| --- | --- |
| F (DFn, DFd) | 11,13 (3, 35) |
| P value | <0,0001 |
| P value summary | **** |
| Are SDs significantly different (P < 0.05)? | Yes |

| **Shapiro-Wilk test** | **Ctrl** | **Aβ** | **Aβ + Oxo** | **Oxo** |
| --- | --- | --- | --- | --- |
| W | 0,8906 | 0,9176 | 0,9506 | 0,9070 |
| P value | 0,2022 | 0,3374 | 0,6753 | 0,2611 |
| Passed normality test (alpha=0.05)? | Yes | Yes | Yes | Yes |
| P value summary | ns | ns | ns | ns |

Figure 5A

| **Brown-Forsythe test** |  |
| --- | --- |
| F (DFn, DFd) | 3,976 (3, 32) |
| P value | 0,0163 |
| P value summary | * |
| Are SDs significantly different (P < 0.05)? | Yes |

| **Shapiro-Wilk test** | **Ctrl** | **Aβ** | **Aβ + Oxo** | **Oxo** |
| --- | --- | --- | --- | --- |
| W | 0,9436 | 0,8595 | 0,9854 | 0,8651 |
| P value | 0,6198 | 0,0947 | 0,9863 | 0,1088 |
| Passed normality test (alpha=0.05)? | Yes | Yes | Yes | Yes |
| P value summary | ns | ns | ns | ns |

Figure 5B

| **Brown-Forsythe test** |  |
| --- | --- |
| F (DFn, DFd) | 0,2360 (3, 36) |
| P value | 0,8707 |
| P value summary | ns |
| Are SDs significantly different (P < 0.05)? | No |
|  |  |

| **Shapiro-Wilk test** | **Ctrl** | **Aβ** | **Aβ + Oxo** | **Oxo** |
| --- | --- | --- | --- | --- |
| W | 0,9010 | 0,9233 | 0,9582 | 0,9638 |
| P value | 0,2248 | 0,3851 | 0,7650 | 0,8280 |
| Passed normality test (alpha=0.05)? | Yes | Yes | Yes | Yes |
| P value summary | ns | ns | ns | ns |

Figure 5C

| **Brown-Forsythe test** |  |
| --- | --- |
| F (DFn, DFd) | 2,151 (3, 32) |
| P value | 0,1132 |
| P value summary | ns |
| Are SDs significantly different (P < 0.05)? | No |
|  |  |

| **Shapiro-Wilk test** | **Ctrl** | **Aβ** | **Aβ + Oxo** | **Oxo** |
| --- | --- | --- | --- | --- |
| W | 0,9169 | 0,8970 | 0,9354 | 0,9481 |
| P value | 0,3673 | 0,2352 | 0,5339 | 0,6695 |
| Passed normality test (alpha=0.05)? | Yes | Yes | Yes | Yes |
| P value summary | ns | ns | ns | ns |

Figure 6A

| **Brown-Forsythe test** |  |
| --- | --- |
| F (DFn, DFd) | 0,9371 (3, 32) |
| P value | 0,4341 |
| P value summary | ns |
| Are SDs significantly different (P < 0.05)? | No |

| **Shapiro-Wilk test** | **Ctrl** | **Aβ** | **Aβ + Oxo** | **Oxo** |
| --- | --- | --- | --- | --- |
| W | 0,9352 | 0,9498 | 0,9427 | 0,9285 |
| P value | 0,5325 | 0,6883 | 0,6107 | 0,4674 |
| Passed normality test (alpha=0.05)? | Yes | Yes | Yes | Yes |
| P value summary | ns | ns | ns | ns |

Figure 6B

| **Brown-Forsythe test** |  |
| --- | --- |
| F (DFn, DFd) | 1,603 (3, 32) |
| P value | 0,2080 |
| P value summary | ns |
| Are SDs significantly different (P < 0.05)? | No |

| **Shapiro-Wilk test** | **Ctrl** | **Aβ** | **Aβ + Oxo** | **Oxo** |
| --- | --- | --- | --- | --- |
| W | 0,9330 | 0,8747 | 0,9089 | 0,8639 |
| P value | 0,5109 | 0,1379 | 0,3081 | 0,1057 |
| Passed normality test (alpha=0.05)? | Yes | Yes | Yes | Yes |
| P value summary | ns | ns | ns | ns |

Figure S1A

| **Brown-Forsythe test** |  |
| --- | --- |
| F (DFn, DFd) | 2,045 (3, 32) |
| P value | 0,1273 |
| P value summary | ns |
| Are SDs significantly different (P < 0.05)? | No |

| **Shapiro-Wilk test** | **Ctrl** | **Oxo 24h** | **Oxo 48h** | **Oxo 72h** |
| --- | --- | --- | --- | --- |
| W | 0,9582 | 0,9039 | 0,9589 | 0,9119 |
| P value | 0,7789 | 0,2753 | 0,7873 | 0,3297 |
| Passed normality test (alpha=0.05)? | Yes | Yes | Yes | Yes |
| P value summary | ns | ns | ns | ns |

Figure S1B

| **Brown-Forsythe test** |  |
| --- | --- |
| F (DFn, DFd) | 1,589 (3, 44) |
| P value | 0,2055 |
| P value summary | ns |
| Are SDs significantly different (P < 0.05)? | No |

| **Shapiro-Wilk test** | **Ctrl** | **Oxo 24h** | **Oxo 48h** | **Oxo 72h** |
| --- | --- | --- | --- | --- |
| W | 0,9349 | 0,9019 | 0,9319 | 0,9490 |
| P value | 0,4349 | 0,1679 | 0,4010 | 0,6231 |
| Passed normality test (alpha=0.05)? | Yes | Yes | Yes | Yes |
| P value summary | ns | ns | ns | ns |

Figure S2A

| **Brown-Forsythe test** |  |
| --- | --- |
| F (DFn, DFd) | 0,3640 (3, 44) |
| P value | 0,7793 |
| P value summary | ns |
| Are SDs significantly different (P < 0.05)? | No |

| **Shapiro-Wilk test** | **Ctrl** | **Oxo** | **Atropine+Oxo** | **Atropine** |
| --- | --- | --- | --- | --- |
| W | 0,9259 | 0,9005 | 0,9407 | 0,9172 |
| P value | 0,3383 | 0,1612 | 0,5078 | 0,2635 |
| Passed normality test (alpha=0.05)? | Yes | Yes | Yes | Yes |
| P value summary | ns | ns | ns | ns |

Figure S2B

| **Brown-Forsythe test** |  |
| --- | --- |
| F (DFn, DFd) | 0,7465 (3, 44) |
| P value | 0,5302 |
| P value summary | ns |
| Are SDs significantly different (P < 0.05)? | No |

| **Shapiro-Wilk test** | **Ctrl** | **Nicotine 24h** | **Nicotine 48h** | **Nicotine 72h** |
| --- | --- | --- | --- | --- |
| W | 0,9574 | 0,8519 | 0,8687 | 0,9401 |
| P value | 0,7465 | 0,0387 | 0,0630 | 0,4990 |
| Passed normality test (alpha=0.05)? | Yes | No | Yes | Yes |
| P value summary | ns | * | ns | ns |

Figure S2C

| **Brown-Forsythe test** |  |
| --- | --- |
| F (DFn, DFd) | 0,1397 (3, 44) |
| P value | 0,9357 |
| P value summary | ns |
| Are SDs significantly different (P < 0.05)? | No |

| **Shapiro-Wilk test** | **Ctrl** | **Nicotine 24h** | **Nicotine 48h** | **Nicotine 72h** |
| --- | --- | --- | --- | --- |
| W | 0,9663 | 0,9787 | 0,8985 | 0,9318 |
| P value | 0,8682 | 0,9781 | 0,1516 | 0,3992 |
| Passed normality test (alpha=0.05)? | Yes | Yes | Yes | Yes |
| P value summary | ns | ns | ns | ns |

Figure S3A

| **Brown-Forsythe test** |  |
| --- | --- |
| F (DFn, DFd) | 0,2976 (3, 39) |
| P value | 0,8269 |
| P value summary | ns |
| Are SDs significantly different (P < 0.05)? | No |

| **Shapiro-Wilk test** | **Ctrl** | **Aβ** | **Aβ+Oxo** | **Aβ+Oxo+**  **Atropine** |
| --- | --- | --- | --- | --- |
| W | 0,9004 | 0,8899 | 0,8709 | 0,9122 |
| P value | 0,2542 | 0,1176 | 0,0797 | 0,2587 |
| Passed normality test (alpha=0.05)? | Yes | Yes | Yes | Yes |
| P value summary | ns | ns | ns | ns |

Figure S3A

| **Brown-Forsythe test** |  |
| --- | --- |
| F (DFn, DFd) | 0,2821 (3, 38) |
| P value | 0,8380 |
| P value summary | ns |
| Are SDs significantly different (P < 0.05)? | No |

| **Shapiro-Wilk test** | **Ctrl** | **Aβ** | **Aβ+Nicotine 100µM** | **Aβ+Nicotine 10µM** |
| --- | --- | --- | --- | --- |
| W | 0,9004 | 0,8899 | 0,8518 | 0,8228 |
| P value | 0,2542 | 0,1176 | 0,0450 | 0,0274 |
| Passed normality test (alpha=0.05)? | Yes | Yes | No | No |
| P value summary | ns | ns | * | * |

**Supplementary Figure** **1**


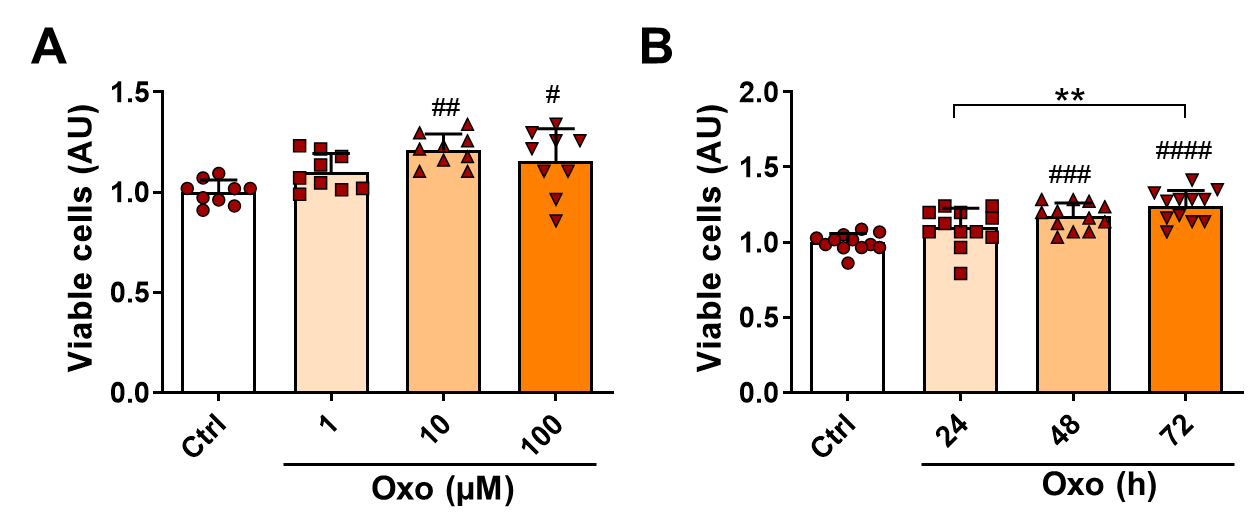


**Figure S1** Oxo neurotrophic effects. A) Dose-effect of Oxo treatment (48 h) on cell viability, evaluated by Trypan blue exclusion method count. B) Time-course of Oxo treatment (10 µM) effects on cell viability, assessed by Trypan blue exclusion method count. Data are plotted as mean and SD. *Post-hoc test*: # p < 0.05, ## p < 0.01, ### p < 0.001, #### p < 0.0001 as compared to control (Ctrl) group; ** p< 0.01

**Supplementary Figure 2**

**
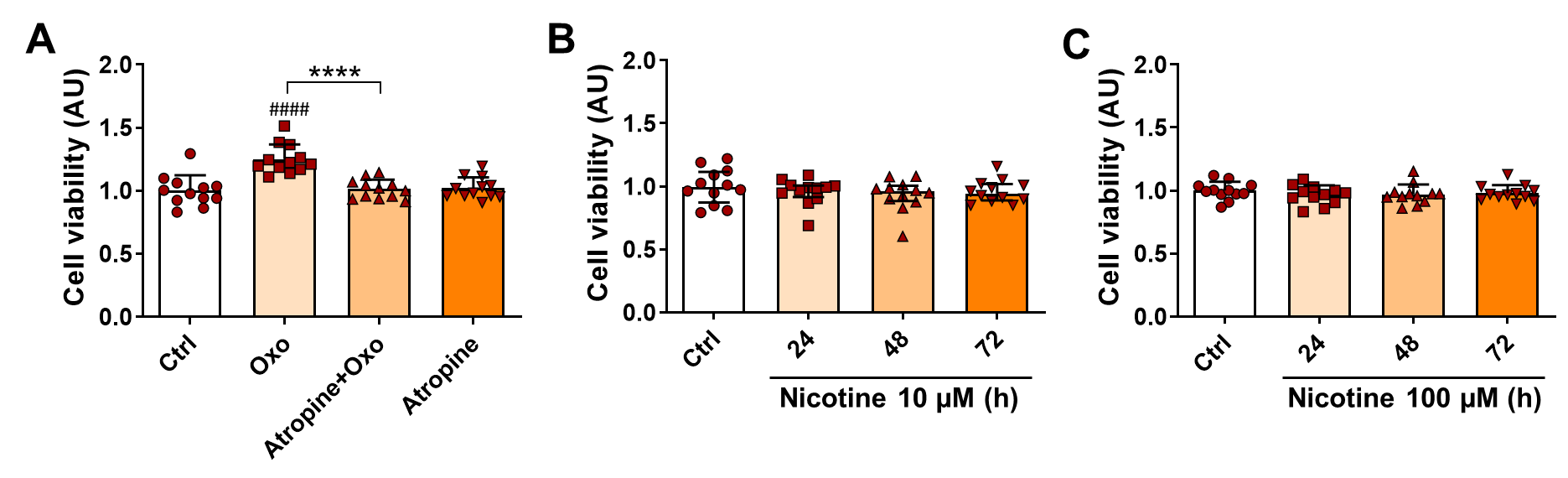
**

**Figure S2** Involvement of mAchRs in Oxo neurotrophic effects. A) Quantification of cell viability by MTT assay in control (Ctrl) cells, cells treated with Oxo (10 µM, 72 h), Atropine (100 µM, 72 h) + Oxo (10 µM, 72 h), and Atropine (100 µM, 72 h). B) Time-course of Nicotine treatment (10 µM) effects on cell viability, assessed by MTT test. C) Time-course of Nicotine treatment (100 µM) effects on cell viability, assessed by MTT test. Data are plotted as mean and SD in A) and C), and as median and interquartile range in B). *Post-hoc test*: #### p < 0.0001 as compared to control (Ctrl) group; **** p< 0.0001

**Supplementary Figure 3**


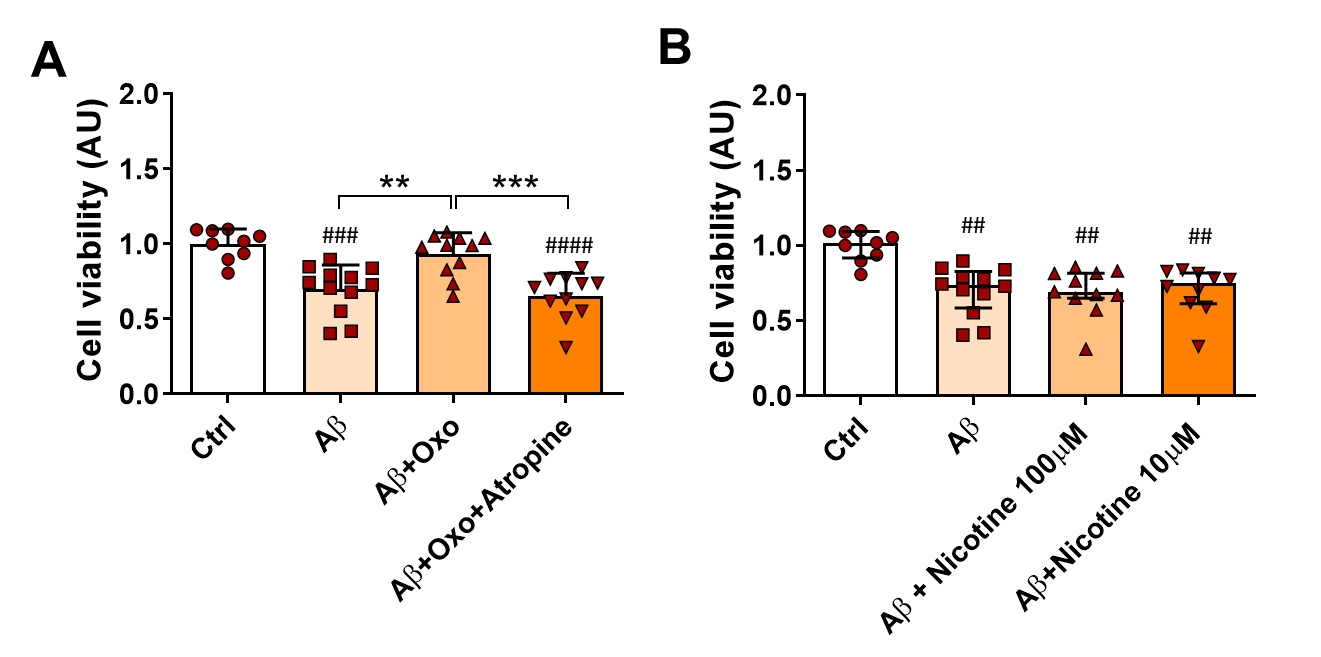


**Figure S3** Involvement of mAchRs in Oxo neuroprotective effects against Aβ-induced cell death. A) Quantification of cell viability by MTT assay in control (Ctrl) cells, cells treated with Aβ_1-42_ (600 nM, 24 h), Aβ_1-42_ (600 nM, 24 h) + Oxo (10 µM, 24 h), and Aβ_1-42_ (600 nM, 24 h) + Oxo (10 µM, 24 h) + Atropine (100 µM, 24 h). B) Quantification of cell viability by MTT assay in control (Ctrl) cells, cells treated with Aβ_1-42_ (600 nM, 24 h), Aβ_1-42_ (600 nM, 24 h) + Nicotine (100 µM, 24 h), and Aβ_1-42_ (600 nM, 24 h) + Nicotine (10 µM, 24 h). Data are plotted as mean and SD in A) and as median and interquartile range in B). *Post-hoc test*: ## p < 0.01, ### p < 0.001, #### p < 0.0001 as compared to control (Ctrl) group; ** p< 0.01, *** p< 0.001
